# Nutrition knowledge and dietary intake in male English Premier League academy players and their key stakeholders

**DOI:** 10.1080/15502783.2026.2715064

**Published:** 2026-09-02

**Authors:** Alexander Levington, Andrew Hulton, Kyriaki Myrissa, Patrick Oxenham, Olivia Coope, Stephen Patterson, Sam Milner, Tilly J Spurr, James Fleming

**Affiliations:** a Faculty of Sport, Technology and Health Sciences, St Mary's University, Twickenham, UK; b Fulham FC, Craven Cottage, London, UK; c Discipline of Nutrition, Exercise, Chronobiology and Sleep, University of Surrey, Guildford, UK; d Blanquerna School of Health Sciences, Ramon Llull University, Barcelona, Spain; e School of Sport, Science and Engineering, University of Chichester, College Lane, Chichester, UK

**Keywords:** Nutrition knowledge, dietary intake, academy footballer

## Abstract

**Background:**

Adequate nutrition knowledge (NK) and dietary intake are important to the development in Youth Development Phase (YDP) academy footballers, helping to fuel sporting performance and promote long-term health. However, these areas remain under-researched within English Premier League (EPL) academy settings. This study aimed to investigate NK and energy intake (EI) in YDP academy players, including key stakeholders' knowledge, within a single Category 1 Academy.

**Methods:**

Eighty-three EPL Category 1 YDP academy players (U11s = 17, U12s = 16, U13s = 8, U14s = 17, U15s = 10, and U16s = 15; 14 ± 2 years), 6 academy coaches, 1 YDP sports scientist and 31 caregivers' knowledge was assessed through the 89-item Knowledge and Sports Questionnaire (NSKQ). Twenty-five EPL Category 1 YDP academy players (U12s: *n* = 8, U13s: *n* = 9, U14s: *n* = 5, and U16s: *n* = 3; 13 ± 2 years) dietary intake was also assessed using an estimated written 7-day food diary collected during the in-season (2023/2024 season, March to May 2023).

**Results:**

Collectively, academy players (33% ± 8%), caregivers (40% ± 13%), and coaches (41% ± 9%) scored poorly on NK. Academy players reported sub-optimal energy intake (1742 ± 314 kcal·day^−1^ and 38.5 ± 11.4 kcal·kg⁻¹·day⁻¹), accompanied by poor intake of daily carbohydrate (<5 g·kg−1·d^−1^), and fiber (<25 g/day) referenced to recommended daily intakes. Daily protein intake (1.7 ± 0.5 g·kg−1·d^−1^) exceeded the recommended intake for adolescent footballers, while fat intake (27% ± 6%) expressed as a percentage of total dietary intake (TDI) was within the guidelines. NK was not correlated with absolute EI, (*r*(23) = –.187, *p* = .371, or EI adjusted to BM, (*r*(23) = .204, *p* = .329.

**Conclusion:**

The results indicated poor NK and suboptimal dietary intake in EPL Category 1 academy players accompanied by low knowledge among stakeholders.

## 1. Introduction

Academy footballers in any English Premier League (EPL) Category 1 academies are divided into three age groups: Foundation Phase (FP) (U9s-U11s), YDP (U12s-U16s), and Professional Development Phase (PDP) (U18s-U23s) [1]. The FP and YDP phases are characterized by rapid growth and maturation alongside the development of behaviors that may influence long-term health outcomes [2]. Good eating behaviors in adolescent footballers require the recommended quantity, high-quality [3,4], and optimal distribution of macronutrients [5], which creates a balanced and varied diet. Adequate dietary intake and nutrition knowledge (NK) are important factors in enhancing athletic performance [6] and promoting long-term health.

It is important to investigate energy intake (EI) and dietary intake in relation to accepted sport-specific guidelines to support physiological adaptations for growth, maturation, and overall performance [5,7,8], as well as long-term health. The energy requirements of academy footballers are met through the consumption of macronutrients such as carbohydrates, fat, and protein [5]. Daily macronutrient intake modulates aspects of performance, recovery, and training adaptation [9]. Academy players are often reported to be under-fueled [10] or have a suboptimal dietary intake of macronutrients, particularly carbohydrates [11,12]. A recent review of youth soccer players (U12 to U19) reported an average energy expenditure of 3134 kcal·day^−1^, similar to that of EPL first-team players, which ranged from 3047 to 4400 kcal·day^−1^ [9]. Studies investigating EPL academy players [10,13–15], European [11,16,17] and non-European [18,19] often report varied dietary intakes (~2114–3380 kcal·day^−1^). However, evidence regarding EI and dietary habits in FP or YDP remains unclear, despite the need to support players in meeting their energy requirements.

NK is among several factors influencing the development of recommended eating behaviors in athletes [7]. In young athletes, inadequate NK has been linked to poor dietary behaviors, including low intake of fiber, fruit, vegetables, dairy, and carbohydrates, as well as suboptimal energy consumption [8–10]. Higher NK has been shown to positively influence food choices and the consumption of fruit and vegetables in seventy-three male state-level championship Brazilian adolescent soccer players (aged 14–19 years) [10], emphasizing the importance of equipping young athletes with the knowledge to make better dietary decisions [7]. However, the relationship between NK and dietary intake is not consistently strong, and NK alone has been proposed to be insufficient to improve dietary habits in athletes [20–22]. Heaney [22] reported that the relationship between NK and dietary intake demonstrates a weak positive association (*r* < .44) in individual and team sport athletes. This indicates that while NK may be an important component of dietary behavior, it is likely only one part of a broader behavioral, environmental, and social context influencing food choices.

Caregivers have a significant influence on adolescents' food choices through caregiver styles and behaviors, such as shared family meals [23,24]. Caregivers' influence occurs via several mechanisms, including the availability of foods in the home, caregiver modeling of eating behaviors, the enforcement of food-related rules and expectations, and the social environment created during mealtimes [24,25]. Together, these factors make caregivers key nutritional gatekeepers, shaping both the types of foods available and the eating norms within the household. For youth athletes specifically, caregivers are responsible for buying, preparing, and providing food, and their nutrition knowledge can directly impact dietary practices, with insufficient knowledge becoming a barrier to optimal nutrition [1]. Therefore, caregivers' dietary habits and feeding practices are vital determinants of children's and adolescents' dietary intake [26].

Current research on the validated nutrition for sport knowledge questionnaire (NSKQ) [27] has shown low NK including male professional Australian footballers (46% ± 13%) [28], female collegiate athletes (45% ± 10%) [29], male elite and sub-elite Gaelic football players (44% ± 12%) [30], caregivers supporting YDP (44% ± 13%) and FP (41% ± 15%) male academy footballers [1]. However, two published investigations with female Australian footballers [31] and male Polish footballers [32] scored “average” (51%) on the 89-item NSKQ instrument [27]. To date, only one study has assessed caregivers' NK in the FP and YDP of United Kingdom (UK) academy players, reporting low scores on the 89-item NSKQ instrument in both Category 1 (45% ± 13%) and Category 2 (39% ± 15%) academies [1]. In a shorter validated version of the NSKQ instrument, known as the abridged nutrition for sports knowledge questionnaire (A-NSKQ) [33], reported poor knowledge in caregivers (43% ± 14%) and host-families (50% ± 12%) of PDP academy players [34].

Adolescent soccer players often receive nutrition guidance from non-specialists within academy settings, such as coaches, physiotherapists and sports science staff, likely due to limited access to qualified sports nutrition practitioners, who frequently work part-time across multiple squads [35]. Consequently, players in the FP and YDP phases may have fewer opportunities for structured, evidence-based nutrition education. Indeed, Carney [35] reported that accredited nutritionists in Category 1 academies delivered substantially more monthly support hours to PDP players (~60–70 h·month^−1^) compared with YDP (~20 h·month^−1^) and FP (~5 h·month^−1^), alongside greater one-to-one support, food provision and supplementation. This disparity in service provision may limit the development of adequate nutrition knowledge and players' ability to make informed dietary choices that support performance, growth and long-term health.

This study aimed to investigate NK and dietary intake in male EPL YDP academy players, including caregivers, and academy coaches' knowledge. A secondary aim was to assess if an association between NK and EI exists in these players. Once NK and EI are acquired in academy settings, appropriate targeted nutrition interventions can be implemented or provide a rationale for further investigations.

## 2. Methods

### 2.1. Study design

In a cross-sectional design, participants' NK was measured using a validated 89-item NSKQ [27]. Academy players' EI and dietary intake were assessed using a 7-day in-season food diary. Participants were recruited from a single Category 1 Academy in the YDP pathway. Players were excluded if playing in the PDP or injured. Testing procedures were conducted over twelve weeks, from March to May 2023, during the 2023/2024 season.

### 2.3 Participants and recruitment

A convenience sample of 83 EPL Category 1 Academy players (U11s: n = 17, U12s: n = 16, U13s: n = 8, U14s: n = 17, U15s: n = 10, and U16s: n = 15; 14 ± 2 years) participated in the completion of the 89-item NSKQ questionnaire. Food diaries were completed by 25 of the 83 players (30%) (U12s: n = 8, U13s: n = 9, U14s: n = 5, and U16s: n = 3; 13 ± 2 years), who completed a 7-day dietary record. All players had been at the academy for longer than 3-years. All caregivers *(*n* = 124)* were invited to complete the 89-item NSKQ questionnaire with 27% accepting (n* = 31,* 41 ± 7-years-old). Fourteen coaches and four sports scientists in the YDP were invited to complete the 89-item NSKQ questionnaire, with 43% and 25% accepting the invitation, respectively. Coaches (n* = 6,* M = 5, F = 1, 36 ± 10-years-old) and the sport scientist (31-year-old male**)** had a minimum of 6 years of coaching experience in academy football. Caregivers and coaches completed written informed consent before participating in the study. Academy players completed an assent form at the beginning of the season, allowing multiple studies within the academy. Assent procedures were age-appropriate, with information sheets and verbal explanations tailored to the players' level of understanding, and opportunities provided for questions before participation. Ethical approval was provided by the ethics sub-committee of St Mary's University, Twickenham (SMU_2022-23_700). The study was conducted in accordance with the Declaration of Helsinki [34,36].

### 2.4. Nutrition knowledge

NK was assessed using the 89-item NSKQ [27]. The NSKQ covers General knowledge and Sports Nutritional knowledge, divided into five sections: weight management (13 items), macronutrients (30 items), sports nutrition principles (13 items), supplements (12 items), and alcohol (8 items). The design of the NSKQ allows for a detailed understanding of specific areas of NK and highlights the knowledge needed to support players' progression through the academy pathways. NK is classified according to the following criteria; (1) “poor” (0%–49%), (2) “average” (50%–65%), (3) “good” (66%–75%), and (4) “excellent” (75%–100%) [27].

Academy players were asked to arrive at the training ground 60 minutes before a designated training session. During the session, a brief introduction was given to all academy players on how to fill in the paper-based questionnaire. Each correct answer was awarded 1 point with no negative scoring; both incorrect and “not sure” were scored as zero. Players were instructed to work independently and not to discuss the questions until everyone had finished. Coaches completed the same questionnaire in a one-to-one session at the academy training ground. Caregivers were sent the same questionnaire online (Version 2; Jisc, Bristol, UK) via the club's network messaging application (Version 28.1.5; Kitman Labs Ltd., Dublin, Ireland) with the same instructions as players.

### 2.5. Dietary intake

Dietary intake was assessed using a 7-day written food diary. Twenty-five Category 1 academy players (13 ± 1 years) were asked to report all food, fluids, and supplements consumed over a 7-day period in season. A 7-day food diary offers a more accurate and comprehensive representation of dietary intake than shorter tracking periods, capturing typical eating habits, reducing daily variability, and highlighting a broader range of foods and patterns [37,38]. Prior to data collection, players and caregivers attended a 15-minute briefing before training on how to complete the food diary. Written instructions and an example entry were provided. Players and caregivers were instructed to estimate portion sizes using household measures (e.g. tablespoons, cups) and packet information where available. Additional guidance was provided on recording brand names, cooking methods, sauces, and condiments. For meals consumed outside the home (e.g. restaurants, school meals, or takeaway foods), participants were asked to record the establishment, meal description, and portion size as accurately as possible. To check the reliability of the food diary analysis, a sample of five caregivers were randomly selected for a phone call on the fourth day of collection to validate the food diaries. The validation used a 24-hour recall (multiple-pass method) procedure (cues for portion size, use of condiments, brands, and cooking methods, etc.) and asked the five caregivers to report on what foodstuffs and fluids the player consumed on the previous day [37]. Following the briefing, players and caregivers were sent a blank food diary via the club's network messaging application (Version 28.1.5; Kitman Labs Ltd., Dublin, Ireland).

### 2.6. Data analysis

Data from all players, caregivers, and club staff were exported to Microsoft Excel (Version 2011), and statistical analysis was performed using the Statistical Package for the Social Sciences (Version 21.0; IBM Corp., Armonk, NY, USA). Food diary data were analyzed through Nutritics software (version 3.74 professional edition, Nutritics Ltd., Co. Dublin, Ireland). The food diary analysis calculated the absolute and relative to BM, EI, macronutrient, saturated fat, and fiber intake across a 7-day week.

The number of fruits, vegetables, and oily fish intake were compared to national guidelines [39,40] directly from the written food diary. Where portion sizes were not given for fruit or vegetable intake, standard UK National Health Service (NHS) guidance was used, with one portion of fresh, frozen or canned fruit or vegetables defined as 80 g, and one portion of dried fruit as 30 g. Oily fish intake was quantified using the standard portions used in dietary analysis software (Nutritics software version 3.74 professional edition, Nutritics Ltd., Co. Dublin, Ireland). All variables were examined for normality according to the skewness and kurtosis parameters (±1.96), the Shapiro–Wilk test and visual inspection of histograms. Correlation analyzes were conducted to assess the relationship between EI and NK. Pearson's correlation (*r*) was used for normally distributed data, while Spearman's rank correlation (r_s_) was applied when normality assumptions were violated, based on Shapiro–Wilk tests and visual inspection of histograms. The type of correlation used is reported alongside each result. The effect size of the correlation was based on small (0.2), medium (0.5), and large (0.8) [41].

A Kruskal–Wallis's test was used to examine the differences in NK between players' age groups. Dunn's post-hoc analysis was used to identify differences between players. Separate Kruskal–Wallis tests were conducted for each NSKQ sub-category (weight management, macronutrients, micronutrients, sports nutrition, supplementation, and alcohol) to examine differences in scores across academy player age groups (U11-U16), caregivers, and coaches. A one-way ANOVA was used to examine differences in NK across participant groups (academy players, caregivers, and coaches). In addition, a one-way ANOVA was used to examine differences in EI, carbohydrates, and protein intake across player age groups. Homogeneity of variances was assessed using Levene's test. If the Levene's test was violated, Welch's statistic was used to present the ANOVA. In the presence of significant main effects, the Games-Howell or Gabriel (for unequal sample sizes) post-hoc analysis was used to identify differences between participants. The data are displayed as mean ± SD; absolute denotes the total daily intake, and relative denotes the point at which the absolute data has been normalized to each participant's body mass. An intraclass correlation coefficient (ICC) using an absolute agreement, two-way mixed-effects model was conducted to assess the level of agreement between players' food diary analysis and caregivers' 24-hour dietary recall. The reliability values were based on (<0.5) poor, moderate (0.5 to 0.75), good (0.75 to 0.9), and excellent reliability (>0.9) [42,43]. Statistical significance was set to an alpha (5%, *p < 0.05).*

## 3. Results

**Figure 1.**
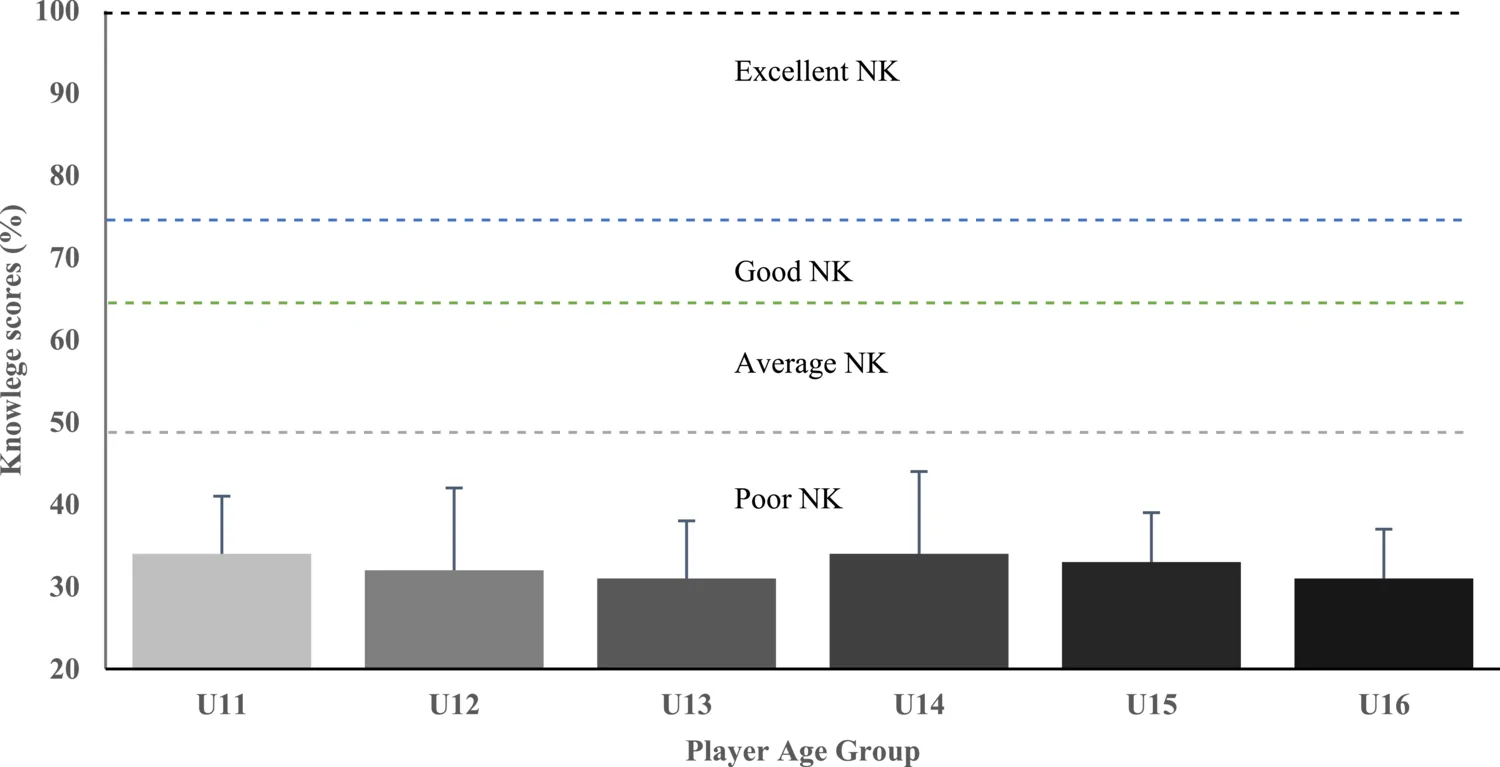
Academy players mean and SD scores in the NK questionnaire. Cut of NK scores illustrated by the dotted line; Grey = poor (0%–49%), Green (50%–65%), Good = Blue (66%–75%), and Excellent (75%–100%) [27].

All participants completed the NSKQ 89-item questionnaire within 60 minutes. Results indicate that all groups have poor NK with an overall mean score of 40% ± 12%. Individual analysis for each group shows players at 33% ± 8%, caregivers at 40% ± 13%, and coaches at 41% ± 9%, highlighting their “poor” knowledge [27]. The sports scientist was removed from the analysis due to sample size *(n = 1)*. A Kruskal–Wallis test showed no significant differences in NK across academy age groups (*H(*5) = 2.740, *p* = .740, ε^2^ = 0.00), with scores ranging from 8% to 48% (Figure 1).

**Figure 2.**
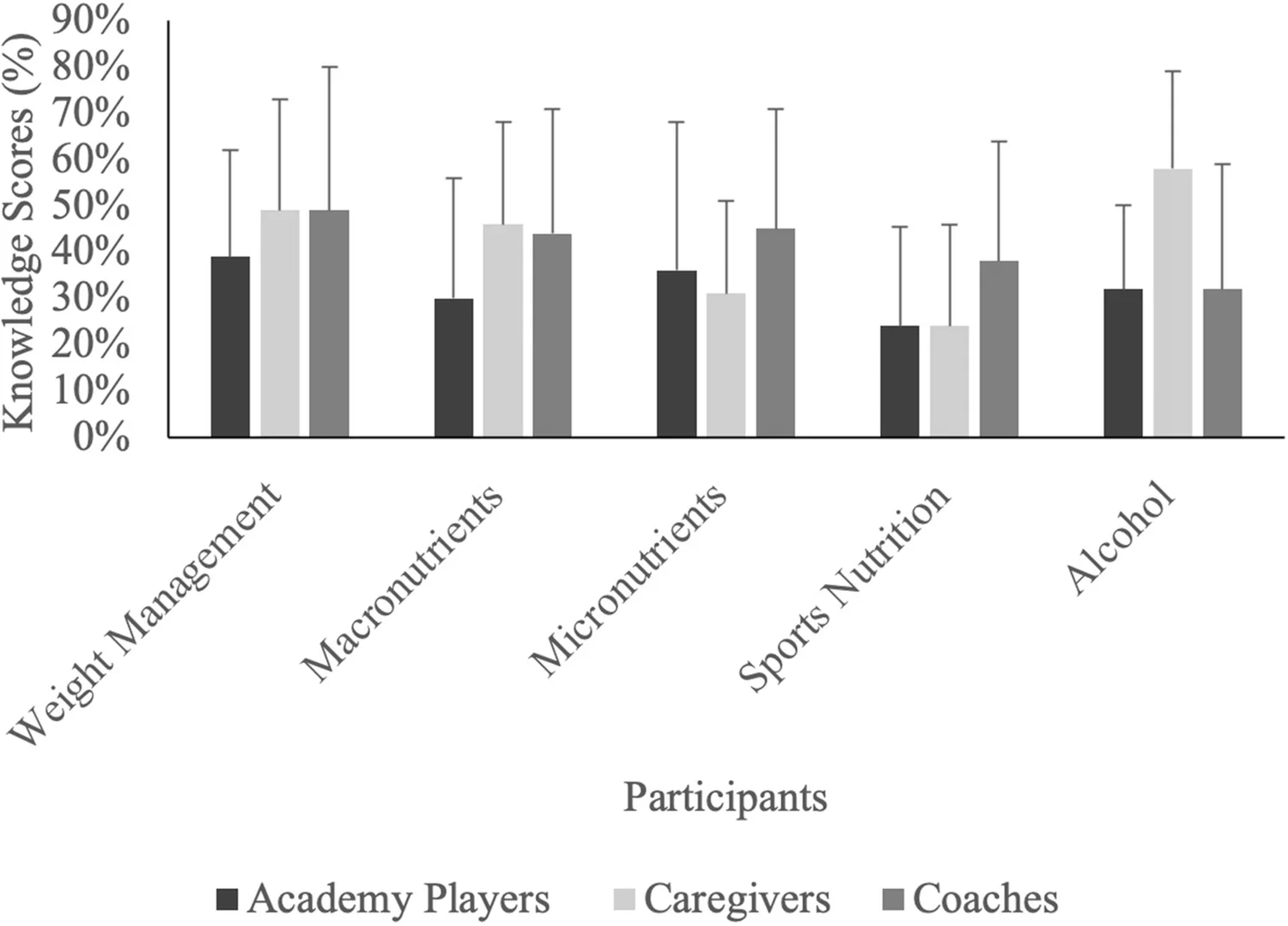
The participants' average knowledge scores in each sub-category.

A one-way ANOVA revealed a significant main effect (*F*(7,112) = 2.278, *p = 0.013,* η^2^ = .13), for NK across academy players, age groups, caregivers, and coaches. However, Gabriel's post-hoc analysis reported no significant differences between age groups. All participants reported that no nutritional support or education had been given to the YDP. A Kruskal–Wallis test reported no significant difference on sub-categories of NK between academy players age groups, caregivers, and coaches including weight management (*H*(7) = 5.357, *p = .617;* ε^2^ = 0.00), macronutrients (*H*(7) = 12.998, *p = .72,* ε^2^ = 0.054), micronutrients (*H*(7) = 6.367, *p = .498,* ε^2^ = 0.00), sports nutrition (*H*(7) = 2.713, *p = 0.910,* ε^2^ = 0.00), supplementation (*H*(7) = 8.279, *p = .309,* ε^2^ = 0.011), and alcohol (*H*(7) = 13.742, *p = 0.056,* ε^2^ = 0.060). See Figure 2 for a visual representation of NK sub-categories.

One-way ANOVA revealed no significant main effect of EI on age groups (*F*(3,21) = 0.874, *p = .470,* η^2^ = .11). A significant main effect was reported when EI was expressed relative to BM (*F*(3,21) = 4.396, *p = .015,* η^2^ = .39) between age groups. The U12s reported significantly greater *(p = .008)* EI when expressed as relative to BM (45.5 ± 9.3 g·kg^−1^·d^−1^) compared to U16s (24.2 ± . 8.6 g·kg^−1^·d^−1^). There was no significant main effect for absolute CHO intake (*F*(3,21) = .718, *p* = .552) or absolute protein intake (*F*(3,21) = 0.128, *p* = .943. There was also no significant main effect for protein intake expressed relative to BM, (*F*(3, 21) = 2.797, *p* = .065). CHO expressed as relative to BM was significantly different between age groups (*F*(3,21) = 3.228, *p = .043,* η^2^ = .32). The U12s (5.7 ± 1.9 g·kg^−1^·d^−1^) had a significantly greater *(p = .027)* intake of CHO expressed as relative to BM compared to the U16s (2.9 ± 1.0 g·kg^−1^·d^−1^).

The majority of academy players did not meet the recommended daily carbohydrate (<6 g·kg^−1^·d^−1^) and daily fiber intake (<25 g/day). Five players met or exceeded the recommended carbohydrate intake (U12s: n = 3, U13s: n = 2). Daily protein (~1.5 g·kg^−1^·d^−1^) intakes were close to the recommended guidelines in all age groups apart from the U12s (1.9 ± 0.4 g·kg^−1^·d^−1^). Total fat intake expressed as a percentage of TDI were within the guidelines (25%–35% of total dietary intake), while daily intake of saturated fats exceeded the recommended guidelines (<11%) in all age groups. No age group consumed the recommended oily fish intake (2 portions·wk^−1^) during the 7-day food diary collection. Further, fruit and vegetable intake in academy players were below the recommended levels (<5-daily servings). Academy players reported no food intake 30-minutes before, during, or within 30-minutes after training. No analysis was performed for U15s due to a small sample size *(n = 1)*. The breakdown of dietary intake is reported in Table 1.

A high degree of reliability was observed between academy players and caregivers for carbohydrate and protein intake (ICC > 0.75), while fat intake demonstrated moderate reliability (ICC = 0.5). No correlation was reported between NK scores and EI, (*r_s_*)(23) = −.268, *p* = .196). Similarly, NK scores showed no correlation when EI was adjusted for BM (*r_s_* (23) = .010, *p* = .962). No correlation for absolute EI was observed with NSKQ sections on weight management (*r_s_* (23) = .165, *p* = .431), macronutrients (*r*(23) = .206, *p* = .324), micronutrients (*r_s_(*23) = .169, *p* = .419), sports nutrition (*r*(23) = −.150), (*p* = .473), supplementation (*r_s_* (23) = −.274, *p* = .185), or alcohol (*r*(23) = .122, *p* = .560).

## 4. Discussion

The aim of the study was to investigate NK with academy players and stakeholders within a single Category 1 Academy, with a secondary aim to examine the relationship between players' NK and EI. It was found that NK was “poor” (<50%) [11,27] in all groups, with multiple categories of NK scored below 50% and only caregivers scoring above 50% in two categories (Weight Management and Alcohol). Academy players reported inadequate EI (1742 ± 313^−1^·day^−1^ kcal∙day^−1^) and CHO (4.7 ± 1.6 g·kg^−1^·day^−1^), although no associations between overall NK, knowledge categories and EI were observed.

The mean EI for the YDP academy (1742 ± 313 kcal∙day^−1^) is lower in the current study than previously reported in Category 1 academies who observed their lowest intakes with U13s to U16s (1903 ± 432 kcal∙day^−1^) [14]. One age group in the current study was below the 30 kcal/kg FFM guideline [9] when EI was expressed as relative to BM (U16 = 24.3 ± 8.6 kcal∙kg^−1^∙day^−1^). Furthermore, the U16s had a significantly lower EI when expressed as relative to BM (24.3 ± . 8.6 kcal∙kg^−1^∙day^−1^) compared to the U12s (45.5 ± 9.3 kcal∙kg^−1^∙day^−1^). Inadequate EI has been associated with reductions in muscle mass, increased susceptibility to injury and illness, heightened risk of overtraining [42], and impaired football performance [12]. Male adolescent athletes may present with symptoms of relative energy deficiency in sport (RED-S), including bone stress injuries, soft tissue damage, anxiety, mood disturbances, and fatigue, when consuming insufficient EI [44]. Prolonged inadequate EI may also disrupt thyroid function, which in adolescents can contribute to impaired concentration, mood swings, depression, altered thermogenesis, and potential cardiovascular complications [45].

Carbohydrate intake was below the recommended levels in most academy players (n* = 20)*, with a mean intake of 4.7 ± 1.6 g·kg^−1^·day^−1^. Five players met or exceeded carbohydrate recommendations (U12s: n = 3; U13s: n = 2). A significant age-related difference was observed, whereby the U12 cohort reported a higher intake (5.7 ± 1.9 g·kg^−1^·day^−1^) compared to the U16 players (2.9 ± 0.9 g·kg^−1^·day^−1^). Carbohydrate intake in the present study is consistent with previous research in male Brazilian adolescent footballers (aged 14–19 years) [20] and a meta-analysis of male junior footballers (aged 10–19 years) [12]. Inadequate carbohydrate intake leads to low glycogen stores, impairing neuromuscular function, decision-making, and technical skills (passing, dribbling, shooting) while increasing fatigue and injury risk in football [46–49].

Low carbohydrate intake has been associated with suppressed thyroid hormone levels, which may negatively impact both health and performance in athletes [45]. Furthermore, inadequate carbohydrate intake is independently associated with poor bone health, increasing the risk of stress fractures and reduced BMD, even when EI is sufficient [18,50]. Academy players in the current study, particularly the U16s, reported sub-optimal carbohydrate intake. While immune function was not assessed in the present study, low carbohydrate availability has previously been associated with increased susceptibility to illness and infection in athletes [51,52]. Therefore, it is important to develop nutritional strategies to ensure adequate EI and carbohydrate intake among academy players to promote athletic performance, but most importantly, long-term health. Focusing on dietary habits, including structured eating patterns and fueling strategies tailored to training demands [53], may represent an effective approach for sports nutritionists within the YDP. This strategy could support players both within and beyond the Category 1 Academy system, ensuring sustained performance, recovery, and long-term health.

Notably, the suboptimal energy and carbohydrate intake observed among U16 players may be attributed to the increased autonomy over food choice and eating behavior that develops during adolescence. Compared to U12 players, who are more likely to experience caregivers' control over meals and eating routines, U16 players have greater independence in their dietary decisions [54]. The onset of puberty marks a significant transition in autonomy, as adolescents begin to assert more independence and decision-making capabilities [55]. This increase in autonomy is influenced by various factors, including biological and social development, societal norms, cultural expectations, and individual experiences [56]. Consequently, during the later stages of adolescence, individuals may gain greater autonomy over dietary choices, including what, when, where, and with whom to eat [57]. However, this developmental shift has been associated with a deterioration in eating practices, as adolescents tend to prioritize convenience, taste, and peer influence over nutritional quality [58]. Therefore, it is possible that the lower carbohydrate intake observed among the U16s in the present study may reflect this increased autonomy and reduced caregiver involvement in food selection, contrasting with the more structured and supervised eating patterns of the U12 group.

Academy players in the current study reported not ingesting carbohydrates during or immediately after training, which would have negatively impacted daily intake. Recommended carbohydrate intake during exercise for young athletes aligns with those established for adults, recommending an intake of 30–60 g·h^−1^ (<1 g·min^−1^) for moderate-to-high-intensity exercise lasting longer than 60 minutes [59]. Recommended carbohydrate intake post-exercise is 1–1.2 g·kg^−1^ BM immediately, followed by 1 g·kg^−1^ BM per hour for four hours [60]. Rigid guidelines may be impractical for academy players, instead, fostering habits of consuming carbohydrate-rich snacks (e.g. sweets, bananas, honey) post-exercise [61] may be more effective.

Daily fiber intake (16 ± 4 g·d^−1^) recorded in academy players (Table 1) were below the national daily recommended intake of 25 g [40]. Insufficient fiber intake in young footballers [10,20,62,63] and adult footballers [64] is commonly reported. Inadequate intake of daily carbohydrates and fruit and vegetables (<2 servings per day) was a contributing factor leading to the academy players' low fiber intake. A diet rich in high-fiber foods reduces the risk of chronic disease, improves bowel function [65] and gut microbiota type, leading to better long-term health outcomes [66]. While sports nutrition guidelines can limit dietary fiber intake around activity [67], it is unlikely that academy players in this study were following sports nutrition guidelines, given the carbohydrate and fruit/vegetable intake reported.

**Table 1.** ​Overview of academy players' mean and standard deviation for dietary intake after a 7-day in-season food diary. ​

Measurement	Individual age group intake	Mean daily intake		Daily recommendation	
Total EI	U12 = 1717 ± 181 kcal·day⁻¹	1742 ± 314 kcal·day⁻¹		n/a	
	U13 = 1768 ± 367 kcal·day⁻¹				
	U14 = 1890 ± 337 kcal·day⁻¹				
	U16 = 1553 ± 395 kcal·day⁻¹				
EI relative to body mass	*U12 = 45.5 ± 9.3 kcal∙kg^-1^∙day^-1^	38.5 ± 11.4 kcal∙kg^-1^∙day^-1^		>30 kcal∙kg^-1^∙day^-1^of FFM/day [9]	
	U13 = 38.5 ± 8.7 kcal∙kg^-1^∙day^-1^				
	U14 = 38.6 ± 12.3 kcal∙kg^-1^∙day^-1^				
	*U16 = 24.3 ± 8.6 kcal∙kg^-1^∙day^-1^				
CHO (g)	U12 = 212 ± 43 g	213 ± 45 g		n/a	
	U13 = 217 ± 56 g				
	U14 = 230 ± 30 g				
	U16 = 186 ± 42 g				
CHO (g·kg^−1^·d^−1^)	U12 = 5.7 ± 1.9 g·kg^−1^·d^−1^	4.7 ± 1.6 g·kg^−1^·d^−1^		6 to 8 g·kg^−1^·d^−1^ [9,68,69]	
	U13 = 4.7 ± 1.2 g·kg^−1^·d^−1^				
	U14 = 4.6 ± 1.2 g·kg^−1^·d^−1^				
	U16 = 2.9 ± 0.9 g·kg^−1^·d^−1^				
Protein (g)	U12: 75 ± 20 g	78 ± 17 g		n/a	
	U13: 81 ± 15 g				
	U14: 77 ± 13 g				
	U16: 77 ± 30 g				
Protein (g·kg^−1^·d^−1^)	U12: 1.9 ± 0.4 g·kg^−1^·d^−1^	1.7 ± 0.5 g·kg^−1^·d^−1^		1.5 g·kg−1·d−1 [9,68,69]	
	U13: 1.7 ± 0.3 g·kg^−1^·d^−1^				
	U14: 1.5 ± 0.4 g·kg^−1^·d^−1^				
	U16: 1.2 ± 0.6 g·kg^−1^·d^−1^				
Fat (g)	U12: 63 ± 11 g	64 ± 15 g		n/a	
	U13: 64 ± 15 g				
	U14: 73 ± 21 g				
	U16: 56 ± 13 g				
Fat (%)	U12 = 28% ± 6%	27% ± 6%		20%–35% [70]	
	U13 = 28% ± 2%				
	U14 = 21% ± 11%				
	U16 = 27% ± 2%				
Saturated fat (g)	U12 = 219 ± 51 g	13 ± 2 g		n/a	
	U13 = 210 ± 16 g				
	U14 = 256 ± 47 g				
	U16 = 188% ± 77 g				
Saturated fat (%)	U12 = 13% ± 3%	13% ± 2%		<10% [70]	
	U13 = 12% ± 2%				
	U14 = 14% ± 1%				
	U16 = 12% ± 2%				
Fiber (g/day)	U12: 16 ± 4 g-per-day	16 ± 4g		25 g/day [39]	
	U13: 18 ± 6 g-per-day				
	U14: 15 g ± 2-per-day				
	U16: 15 g ± 4 g-per-day				
Fruit and vegetables	U12: 5 ± 1-per-day	3 ± 1-serving per week		Five Daily Servings [39]	
	U13: 4 ± 2-per-day				
	U14: 2 ± 1-per-day				
	U16: 1 ± 0-per-day				

^*^ Significantly different between U12 and U16 (*p* < 0.05).

EI = energy intake.

Daily protein intake was marginally above sport nutrition recommendations (1.7 ± 0.5 g·kg^−1^·d^−1^) while the U16s were at the lower end of the recommendations 1.2 g·kg^−1^·d^−1^ (U16s). These results are comparable to previous investigations within Category 1 academy players [10,14]. As adolescents experience large increases in FFM during maturation, meeting protein requirements is fundamental to growth and maturation [9]. However, in the academy players that consume inadequate energy, protein availability will be reduced to maintain homeostasis of blood glucose, compromising proteins' primary functions [5]. Fat intake (27% ± 6% of TDI) aligned with nutritional guidelines, while saturated fat (13%) exceeded the national guidelines (<11%) [70]. The reported daily saturated fat intake in the current study was higher than previously recorded in adolescent footballers [20,62].

The present results in academy players are similar to those reported in studies where researchers have adopted the NSKQ with collegiate athletes [12], Australian footballers [28,29] and Gaelic footballers [30]. Investigating NK between age groups is of interest, and current findings illustrate no difference (U11s: 34% ± 7%, U12s: 32% ± 10%, U13s: 31% ± 7%, U14: 34% ± 11%, U15s: 33% ± 6%, and U16s 31% ± 6% scores, Figure 1). This suggests that NK is consistently low across developmental stages within the academy system, with no evidence of improvement as players progress through the age groups. When these results are viewed alongside the UK Department for Education's “Food Teaching in Secondary Schools” framework [71], which emphasizes knowledge related to health, food preparation, and dietary recommendations, these results indicate that while players may be exposed to some health-based nutrition education in school, this does not appear to translate into broader NK domains. Consequently, there is a clear need for academy-based education that integrates health and performance nutrition to equip players with the knowledge to support long-term health and athletic development.

Despite the importance of nutrition education, the present study found no association between NK and EI, suggesting that knowledge alone may not be sufficient to influence dietary intake in academy players. Food choice in adolescent athletes is influenced by a range of behavioral, environmental, and social factors beyond knowledge alone, including food availability, caregiver influence, time constraints, convenience, taste preferences, and the wider food environment [21,22]. In younger age groups, caregivers largely determine food provision, whereas in older age groups, increasing autonomy may lead to greater reliance on convenience foods and irregular eating patterns. Indeed, weak associations between NK and dietary intake may be explained by other behavioral factors such as motivation, habits, and beliefs about the consequences of dietary behaviors [20]. In academy football settings, qualitative research has identified performance implications and role modeling as key enablers of positive dietary behaviors [72]. Performance implications refer to players being more likely to adopt positive dietary behaviors when they perceive a direct link between nutrition and performance outcomes such as training performance, match performance, body composition, and selection [72]. Role modeling refers to the influence of coaches, senior players, and first-team players, where younger players observe and adopt the dietary behaviors and attitudes of those they aspire to emulate [72]. Therefore, improving dietary intake among academy players may require a broader approach that considers the home environment, caregiver support, the influence of coaches and senior players, and the wider academy food environment, rather than focusing solely on improving players' NK.

Caregivers also reported “poor” NK (40% ± 13%), which aligns with previous research using the NSKQ among EPL FP and YDP caregivers (41% ± 15%) [1]. This is a particularly important finding, as caregivers in the YDP play a central role in shaping players' food environments and dietary behaviors [1,34]. Caregivers are typically responsible for purchasing food, meal planning and preparation, providing lunch, arranging transport-related snacks, and the overall structure of eating occasions throughout the day [73]. As such, their influence extends beyond knowledge alone to include food availability at home, modeling of eating behaviors, reinforcement of mealtime routines, and the development of attitudes and relationships with food [24–26]. In practice, even when players possess some NK, their ability to apply it may be constrained by the foods available, family routines, financial considerations, time pressures, and the broader home food environment. This may be especially relevant in academy settings, where training schedules, school commitments, and travel may increase reliance on caregivers to organize meals and snacks around activity [1]. Therefore, poor caregiver NK may represent an important barrier to optimal dietary intake among academy players, particularly in younger age groups who are less autonomous in food choices. Previous research has also documented limited nutrition support for caregivers within EPL academy systems [1,35], which may further reduce caregivers' ability to support evidence-based food choices and eating habits in developing players. Collectively, these findings highlight that future nutrition interventions in academy football should not focus solely on players but also target caregivers as key agents within players' nutritional environments.

In the present study, participants reported an absence of nutritional support in the FP and YDP provided from the club despite caregivers holding a significant influence on food choices in adolescents through parenting styles, and behaviors such as eating together [24]. Academy coaches also reported poor NK (41%), ranging from 33%–49%. These findings align with previous research reporting low NK in coaches from several sports, such as UK hockey and netball [74]. The current study adds to a growing body of evidence that coaches lack adequate NK, highlighting the need for additional nutritional support and training. Considering these and other published findings [1,35], the EPL should review the current policies within the EPPP strategy to reflect the importance of nutrition and attempt to enhance players' and stakeholders' NK. Furthermore, at the time of this study, the academy did not employ a nutritionist. Therefore, it is unsurprising that low NK is present among players, caregivers, and coaches. Indeed, nutrition in EPL academies is not seen as equal value to other disciplines (sport science and medical) [35]. A well-resourced full-time academy nutritionist may not only improve knowledge in players but could potentially have a positive influence on the surrounding key stakeholders.

Despite being a novel study and providing new insights into academy YDP players, there are limitations to consider. The first limitation is that these results represent a single EPL Category 1 Academy, but the methods are reproducible in other academy settings, permitting future comparisons in the area. Secondly, the present study did not explore the influence of dietary habits on match days or away travel. Instead, the study captures 7 days of in-season training only and should be considered a snapshot of EI and dietary intake in this academy setting. The inclusion of matches may indicate varied dietary habits, as matches often take place under diverse environmental conditions, and travel schedules may disrupt dietary habits and sleep patterns, potentially affecting players' diets. Thirdly, the study used a 7-day self-reported food diary, which is subject to several well-documented limitations, including underreporting, inaccurate portion-size estimation, and reporting bias [75]. Self-reported dietary assessment methods are known to be prone to both under- and overestimation of intake, and large inter-individual variability has been observed when estimating nutrient intake in athletic populations, suggesting that such methods may be more appropriate for estimating intake at the group level rather than the individual level [76].

In addition, practitioners' interpretation of food diaries and photographs can introduce additional variability in nutrient analysis, particularly when meals are complex. In addition, dietary assessment methods such as food diaries are prone to dietary reactivity, whereby participants may alter eating behaviors when intake is being recorded, often leading to healthier food choices or reduced intake of discretionary foods. This may be particularly relevant for adolescent athletes, who may adjust their intake to align with perceived expectations of coaches, caregivers, or researchers. National data highlights the magnitude of this limitation, with the UK National Diet and Nutrition Survey (2019–2023) estimating that up to 30% of reported intakes may be underreported, particularly for energy and discretionary foods [40]. Consequently, there may be a need to develop and implement more robust methodologies to assess dietary intake in youth athletes to enable a more accurate understanding of their nutritional status.

In conclusion, this is the first study to investigate NK and EI in male Category 1 EPL YDP academy players, including caregivers and coaches. Importantly, no association was observed between NK and dietary intake among players, highlighting that knowledge alone may be insufficient to influence eating behaviors in this population. This study highlights the need for future interventions in academy players for improvements in NK and EI to support long-term health and progression through the YDP. Specifically, there is a clear case for well-resourced, full-time nutritionists, structured nutrition education for players and stakeholders, and age-appropriate strategies to support healthy eating habits, particularly as players gain autonomy during adolescence. The findings of the present study may be a consequence of the limited provision of structured nutrition support within the academy setting. This observation highlights the importance of embedding appropriately qualified, full-time nutrition practitioners within academies. Ensuring that such roles are well resourced is essential, not only supporting players' nutritional practices, but also to provide evidence-based guidance to caregivers and coaches who act as key stakeholders in the players' developmental environment.
